# Effect and neurophysiological mechanism of acupuncture in patients with chronic sciatica: protocol for a randomized, patient-assessor blind, sham-controlled clinical trial

**DOI:** 10.1186/s13063-018-3164-8

**Published:** 2019-01-16

**Authors:** Koh-Woon Kim, Kyungmo Park, Hi-Joon Park, Geon-Ho Jahng, Dae-Jean Jo, Jae-Heung Cho, Eun-Mo Song, Woo-Chul Shin, Ye-Ji Yoon, Soo-Jeon Kim, Seulgi Eun, Mi-Yeon Song

**Affiliations:** 1 Department of Korean Rehabilitation Medicine, College of Korean Medicine, Kyung Hee University, 26 Kyungheedae-ro, Dongdaemun-gu, Seoul, 02447 Republic of Korea; 2 East-West Medical Research Institute, Kyung Hee University, 23 Kyungheedae-ro, Dongdaemun-gu, Seoul, 02447 Republic of Korea; 3 Department of Biomedical Engineering, Kyung Hee University, 1732 Deogyeong-daero, Giheung-gu, Yongin-si, Gyeonggi-do 17104 Republic of Korea; 4 Studies of Translational Acupuncture Research, Acupuncture and Meridian Science Research Center, Kyung Hee University, 26 Kyungheedae-ro, Dongdaemun-gu, Seoul, 02447 Republic of Korea; 5 Department of Radiology, Kyung Hee University Hospital at Gangdong, College of Medicine, Kyung Hee University, 892 Dongnam-ro, Gangdong-gu, Seoul, 05278 Republic of Korea; 6 Department of Neurosurgery, Spine Center, Kyung Hee University Hospital at Gangdong, College of Medicine, Kyung Hee University, 892 Dongnam-ro, Gangdong-gu, Seoul, 05278 Republic of Korea

**Keywords:** Chronic sciatica, Acupuncture, Effect, Functional magnetic resonance imaging

## Abstract

**Background:**

Sciatica is a relatively frequent illness that easily becomes a chronic and relapsing condition. Although numerous systematic reviews have analyzed various therapies for sciatica, the validity of their included studies is limited. Considering the limitations of conventional treatment options for sciatica, acupuncture is a possible option; however, evidence supporting its efficacy and mechanism in patients with sciatica is lacking. The aim of this proposed protocol is to investigate the effect and neurophysiological mechanism of acupuncture in patients with chronic sciatica.

**Methods/design:**

This study is a randomized, patient-assessor blind, two-arm, parallel, non-penetrating, sham-controlled clinical trial. Eligible participants will include adults (aged 19–70 years old) with a clinical diagnosis of chronic sciatica (40 mm or more of a 100-mm visual analog scale (VAS) for bothersomeness) blinded to the treatment received. Patients will be randomly allocated into the acupuncture treatment group (manual acupuncture plus electroacupuncture (EA), *n* = 34) or the sham acupuncture control group (sham acupuncture plus placebo EA without electrical stimulation, *n* = 34). Groups will receive treatment twice a week for a total of eight sessions over 4 weeks. Functional magnetic resonance imaging will be implemented at baseline and endpoint to investigate the mechanism of acupuncture. The primary outcome measure is the VAS for bothersomeness and secondary outcomes include the VAS for pain intensity, Oswestry Disability Index, EuroQol 5-Dimension, Coping Strategy Questionnaire, Beck’s Depression Inventory, and State-Trait Anxiety Inventory. Adverse events will be assessed at every visit.

**Discussion:**

The results of this trial (which will be available in 2020) should provide important clinical evidence for the effect of acupuncture and demonstrate how acupuncture can be helpful for the treatment of chronic sciatica.

**Trial registration:**

ClinicalTrials.gov, ID: NCT03350789. Registered on 15 November 2017.

**Electronic supplementary material:**

The online version of this article (10.1186/s13063-018-3164-8) contains supplementary material, which is available to authorized users.

## Background

Sciatica is a type of neuropathic pain that includes aching and sharp pain components that radiate from the buttock downward along the course of the sciatic nerve [1, 2]. Of all patients suffering from sciatic pain, 85% of cases are associated with a disk disorder [3]. Symptoms of sciatic pain are frequent with the highest incidence of 40% and the pain easily progresses to a chronic and relapsing stage as disturbances anywhere along the course of the sciatic nerve can cause sciatica [4, 5]. Although numerous systematic reviews and guidelines have analyzed and compared various forms of treatment for sciatica [6–8], the outcomes and the cost of care have remained unchanged for more than a decade [9]. A systematic review concluded that evidence is conflicting regarding the long-term benefits of various strategies for chronic sciatica treatment despite the fact that these same treatments showed some support for short-term pain relief [10]. Recently, investigators have applied neuroimaging studies that explored changes in morphology and functional connectivity of the brain to gain a better understanding of pain and the pain-killing mechanism employed by the nervous system [11–14]. Especially, neurophysiological studies for chronic pain are increasingly important, where the pain continues in spite of no stimulation or irritation and the condition is accompanied by changes in neuroplasticity and sensitization of the nervous system [15–19].

Considering the limitations of conventional treatment options and the importance of understanding the neurophysiological mechanism in chronic pain, acupuncture could be a complementary and alternative treatment option for patients with chronic sciatica. Qin et al. [20] have shown the benefits of acupuncture in patients with sciatica through a systematic review of randomized controlled trials. While the results were affirmative, many of the included trials were of poor quality. Several studies on neuroimaging showed some changes in morphology and functional connectivity of the brain in patients with neuropathic pain such as from herniated intervertebral disk (HIVD) and sciatica [21–26], indicating decreased functional connectivity of the dorsolateral prefrontal cortex (DLPFC) and anterior cingulate cortex (ACC) in chronic sciatica [26]. One study demonstrated that acupuncture treatment for 4 weeks was effective in relieving clinical pain in patients with chronic low back pain (LBP), which correlated with a reverse in the abnormal default mode network (DMN) [27]. Another study showed that 10 acupuncture treatments recovered decreased functional connectivity of DLPFC and ACC in patients with chronic sciatica [26]. These results suggested the possible neurophysiological mechanism of acupuncture for chronic LBP and sciatica in the resting network state; however, these are only a part of the complex mechanism of pain and pain mitigation. Furthermore, few clinical trials have examined the effect of acupuncture on the parameters of emotion and coping strategy with neuroimaging in chronic sciatica patients, even though emotional state, perception, modulation, and chronification are extremely important part in chronic pain [28, 29].

Here, we propose a randomized, patient-assessor blind, sham-controlled study of patients with chronic sciatica with a predetermined sample size and appropriate follow-up following the Consolidated Standards of Reporting Trials (CONSORT) reporting guidelines [30] and Standards for Reporting Interventions in Clinical Trials of Acupuncture (STRICTA) recommendations [31]. This protocol will help investigate the efficacy of acupuncture on chronic sciatica from the perspective of neuroimaging studies using functional magnetic resonance imaging (fMRI) with parameters of emotion and coping strategy to better understand the neurophysiological mechanism of acupuncture on chronic sciatica.

## Methods/design

### Objective

We hypothesize that acupuncture may have a better effect relative to good tolerance in patients with chronic sciatica than sham acupuncture. The aim of this study is to compare the efficacy and safety of acupuncture versus sham acupuncture on bothersomeness, pain, and functional dysfunction due to chronic sciatica. Additionally, neuroimaging studies using fMRI with parameters of emotion and coping strategy will be conducted to better understand the neurophysiological mechanism of acupuncture on chronic pain and related emotional state, perception, modulation, and chronification.

### Study design

**Fig. 1:**
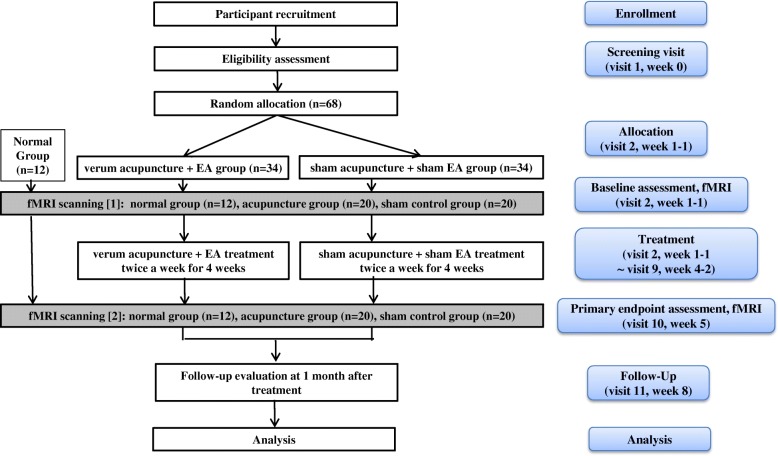
The study flow chart

**Fig. 2:**
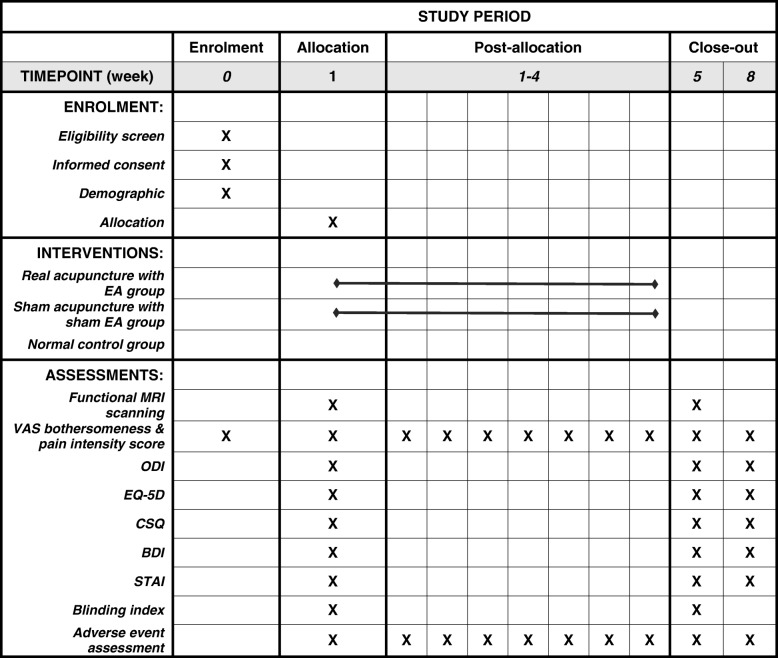
Standard Protocol Items: Recommendations for Interventional Trials (SPIRIT) Figure of enrollment, interventions, and assessments. *EA* electroacupuncture, *VAS* visual analog scale, *ODI* Oswestry Disability Index, *EQ-5D* EuroQol five-dimensional questionnaire, *CSQ* Coping Strategy Questionnaire, *BDI* Beck’s Depression Inventory, *STAI* State-Trait Anxiety Inventory

This clinical research protocol is designed to assess the effect and mechanism of acupuncture for chronic sciatica on pain and associated emotional disorder and will be processed with a randomized, two-arm, parallel, patient-assessor blinded, and non-penetrating sham-acupuncture controlled study. The trial will be performed in the Korean Medicine Hospital of Kyung Hee University at Gangdong in Korea. All participants will be enrolled through voluntary participation and written informed consent will be obtained in accordance with the Declaration of Helsinki and the Guidelines for Good Clinical Practice. Interventions for a given trial participant may be discontinued at any time during the trial based on participant request to discontinue or in cases of significant clinical worsening as judged by Doctors of Korean Medicine (DKMs). This protocol is registered with the U.S. National Institutes of Health Clinical Trials registry (NCT03350789). Eligible participants will be randomly allocated into one of two groups at a 1:1 allocation ratio, blinded to the group allocation, and receive treatment for 4 weeks. The experimental group will undergo real acupuncture treatment (verum manual acupuncture plus electroacupuncture (EA)) and the control group will undergo sham acupuncture treatment (acupuncture without skin penetration plus EA without electrical stimulation) twice a week. The effect on parameters related to bothersomeness, pain, and emotional disorder will be assessed at baseline and endpoint. The fMRI scanning will be conducted for 52 subjects (20 among the 34 experimental subjects, 20 among the 34 sham control subjects, and an additional 12 normal control subjects without any intervention; the normal control group of 12 subjects is for the analysis of fMRI data, not for the main analysis of the primary outcome, the VAS for bothersomeness as they do not have chronic sciatica and do not receive any interventions. They just serve as the normal control with fMRI scans performed twice) at baseline and endpoint. Outcome assessment and statistical analyses will be performed by professionals blinded to the assignment of participants to either the real or sham acupuncture groups. The study flow chart is presented in Fig. 1 and the trial timetable is depicted in Fig. 2.

### Participants

#### Inclusion criteria

A total of 68 patients (with an additional 12 patients as the normal control group for the fMRI data analysis) will be recruited through advertisements in local newspapers, monthly hospital magazines, on the hospital website, and on bulletin boards to achieve adequate enrollment and reach target sample size:

1. Participants from 19 to 70 years of age
2. Participants with clinical diagnosis of chronic sciatica (pain lasting 3 months or more)
3. Participants with a bothersomeness score on the VAS ≥ 40 mm
4. Participants who have voluntarily agreed to participate in the study and have signed written informed consent

#### Exclusion criteria

1. Participants previously having spinal surgery during the last 6 months
2. Participants previously or currently diagnosed with specific severe diseases resulting in sciatica (e.g., malignant tumor, spinal infection, inflammatory spondylitis)
3. Participants with progressive neurological deficit or severe neurological signs
4. Participants currently having other chronic diseases that could disturb the effect of treatments and the results of the study, including cardiovascular diseases, autoimmune diseases, renal diseases, diabetic neuropathy, dementia, epilepsy
5. Participants for whom acupuncture/EA may potentially be inadequate or unsafe (e.g., hemorrhagic diseases, anticoagulant medication, severe diabetes mellitus vulnerable to infections, serious cardiovascular diseases/pacemaker, metal materials in the interior of the body)
6. Participants who are currently pregnant or planning to become pregnant
7. Participants with a history of severe mental illness
8. Participants currently participating other clinical trials
9. Participants previously or currently receiving acupuncture or EA treatment for sciatica or taking medicines that might affect pain symptoms (e.g., corticosteroids, narcotics, non-steroidal anti-inflammatory drugs (NSAIDs)) or considered to be inadequate by the investigators during the last week (but they could be included only after going through 2 weeks of the wash out period)
10. Participants lacking capacity to give informed consent
11. Participants for whom MRI scanning may potentially be inadequate or unsafe (e.g., claustrophobia, metal materials in the interior of the body)
12. Other reasons for exclusion in this trial, including unwillingness to comply with the study protocol or inability to complete the study-related questionnaires by themselves or with assistance, as assessed by researchers

### Randomization, allocation concealment, and blinding

Study participants who meet the eligibility criteria will be randomly assigned to two groups (the acupuncture treatment group or the sham acupuncture control group) in a 1:1 ratio at the second visit after signing written informed consent. Randomization will be conducted using a computer-generated random allocation sequence using SAS 9.4 (SAS Institute Inc., Cary, NC, USA) by a statistician with no clinical involvement in this trial and the random code (maintained in a sealed envelope) will be kept by a clinician who will not contact patients. Blocked randomization will be employed to ensure balance within the two groups.

Subject details will be recorded and a treatment arm and randomization number will be allocated to the subject. The randomization code will be released in the order of sequence after the participants are recruited for the trial and all baseline measures will be taken to ensure allocation concealment. The practitioners will be aware of the allocation arm while the subjects, outcome assessors, and statisticians performing the data analysis will be blinded to the treatment allocation [32]. The blinding index of the acupuncture and sham acupuncture treatments [33], previous experience and knowledge of acupuncture treatment [34], and needle sensation during acupuncture treatment [35] will be evaluated after the first acupuncture treatment, while the credibility and expectancy test will be assessed before the first treatment at baseline.

### Education on study procedure standardization

We developed standard operating procedures (SOPs) for the entire trial and researchers will participate in clinical trial training according to their individual roles based on the SOPs. The process of the study, visit schedule notification, and financial compensation will be explained to participants to ensure their adherence to the protocol.

### Intervention

Both real and sham acupuncture groups will receive a total of eight acupuncture sessions. At the time of the first acupuncture session, all participants will be given an “Exercise Manual for Patients with Sciatica” from the rehabilitation clinic of the Korean Medicine Hospital of Kyung Hee University at Gangdong. More detailed procedures according to the STRICTA are attached in Additional file 1.

#### Acupuncture treatment group

The acupuncture group will receive manual acupuncture plus EA treatment at the same time twice a week for 4 weeks, which is the common approach used by DKMs in South Korea today. The predefined acupuncture points will be carefully selected by a process of consensus with participating DKMs who all specialize in Rehabilitation Medicine of Korean Medicine on the basis of a literature review regarding acupuncture for LBP and sciatica. The seven essential acupuncture points selected are as follows: unilateral (the affected side) *Huantiao* (GB30) for manual acupuncture and *Jiaji* (EX-B2) points at L4 and L5, *Shenshu* (BL23), *Dachangshu* (BL25), *Weizhong* (BL40), and *Yanglingquan* (GB34) for EA. To make the acupuncture treatment reflect an ordinary clinical practice condition, additional individualized manual acupuncture points of 8 or less (a total of 15 or less acupuncture points with the seven essential points) will be chosen by the practitioners among the predefined group of optional acupuncture points categorized according to the three types of meridian patterns identification (Gallbladder meridian pattern, Bladder meridian pattern, and Mixed pattern) [36].

Manual acupuncture treatment will be administered using disposable sterile stainless-steel needles (40 mm × 0.25 mm; Dong-bang Acupuncture Inc., Seoul, Korea) to the acupuncture points mentioned above with the aid of the tube of sham acupuncture needle device. The needles will be inserted perpendicular to a depth of 5–20 mm with the patient lying face down after skin sterilization, followed by bidirectional rotation to induce *Deqi* sensation and left in place for 15 min.

At the same time, the six needles inserted to the predefined EA points will be connected to the ES-160 electrical stimulator. Electrical stimulation will consist of a biphasic waveform current, which utilizes alternating interrupted waves and continuous waves at 50 Hz in a triangular form and a compressional wave [37] for 15 min.

#### Sham acupuncture control group

The sham acupuncture group will receive non-penetrating sham acupuncture as control for manual acupuncture plus placebo acupuncture without electrical stimulation as the control for EA at the same time twice a week for 4 weeks.

Sham acupuncture treatment will be administered using non-penetrating disposable sterile stainless-steel sham needles (40 mm × 0.25 mm) as described by Park et al. (AcuPrime Co., Ltd., Exeter, UK) [38] to a total of 15 or less predefined non-traditional acupuncture points: 2 cm lateral to the each acupuncture point of the acupuncture group. Except for the use of a semi-blunted needle, the technique will be the same as performed in the acupuncture group.

At the same time, the six sham needles inserted to the predefined non-traditional EA points (2 cm lateral to each EA point of the real acupuncture group) will be connected to the ES-160 dummy device that is designed to have no electrical stimulation through insulation of electrodes and be left in place for 15 min.

#### Practitioner background

The acupuncture and sham treatments will be conducted by DKMs licensed by the Korean Ministry of Health and Welfare who specialize in Rehabilitation Medicine of Korean Medicine with at least 3 years of clinical experience. They have studied acupuncture for more than 10 years and graduated from a university of Korean Medicine. They will be required to take the educational course to strictly adhere to the study protocol and be familiar with administering study treatments. All practitioners will undergo intensive and customized training for a full understanding of the sham acupuncture procedure and will be trained to administer acupuncture using a sham needle device. The techniques for the entire treatment procedure will be standardized between practitioners.

### fMRI scanning procedure

fMRI data will be acquired using an Ingenia 3.0 Tesla MRI scanner (Philips Medical System, Best, The Netherlands) equipped for echo planar imaging with an eight-channel head coil. For each of the fMRI series, the blood-oxygenation-level-dependent (BOLD) signal will be acquired using a whole-brain T2-weighted gradient echo pulse sequence (time to repetition [TR]/time to echo [TE] = 2000/35 ms, flip angle = 60°, 80 × 80 acquisition matrix, field of view (FOV) = 230 × 230 mm^2^, voxel size = 3 × 3 × 4 mm^3^, 34 inter-leaved axial slices) will be used. In addition to the fMRI data, we will collect structural data using a three-dimensional T1-weighted (3D-T1W) turbo field echo (TR/TE = 9886/4.59 ms, flip angle = 8°, FOV = 256 × 256 mm^2^, voxel size = 1 mm isotropic) to evaluate brain abnormalities and to analyze fMRI data. The diffusion tensor imaging (DTI) acquisition will be performed in the axial plane and will be obtained with the following imaging parameters: 32 directions, 52 contiguous slices each 2.2 mm thick without gaps between slices, TR 5260 ms, TE 64 ms, factor-b 0 and 800 s/mm^2^, 104 × 104 acquisition matrix, SENSE factor 2.5, FOV = 228 × 228 mm^2^. Participants will be asked to lie supine in the scanner while wearing earplugs to attenuate the gradient noise.

**Fig. 3:**
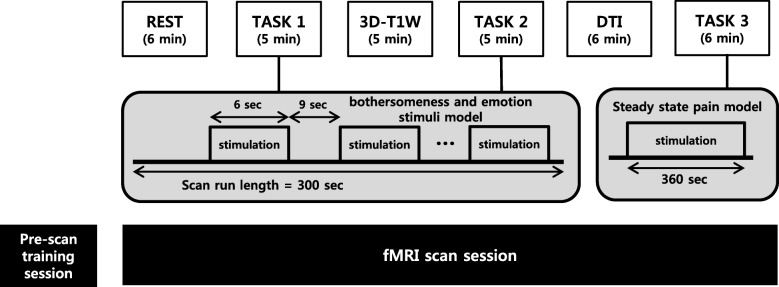
Functional magnetic resonance imaging experiment paradigm (*REST* steady-state resting run; *TASK 1/2* block design bothersomeness and emotion stimulus run; *3D-T1W* structural image acquisition using 3-dimensional T1-weighted turbo field echo; *DTI* diffusion tensor imaging; *TASK 3* low-back-extension pain-model steady-state run)

Subjects will participate in a pre-scan training session that is intended to familiarize them with the electrical stimulator used in TASK 1 and 2 and the computerized cuff-pressure algometry used in TASK 3 and to define their individual pain thresholds, followed by the imaging session, consisting of the REST scan run (6 min), TASK 1 (5 min), 3D-T1W (5 min), TASK 2 (5 min), DTI (6 min), and TASK 3 (6 min) in sequence. The entire imaging session procedure is summarized in Fig. 3.

1. In the training session, participants will be introduced to the Stimuplex® HNS 12 (B. Braun Melsungen AG, Melsungen, Germany) electrical stimulator-induced 3 to 6-s bothersome stimuli and rating procedures for the bothersomeness and emotion stimulation model scan run (TASK 1 and 2). They will be also trained to pain induced by computerized cuff-pressure using the Hokanson® E20 (AG101, Bellevue, WA, USA) rapid cuff inflator system and rating procedures for the low-back-extension (LBE) pain-model scan run (TASK 3). Participant individual bothersomeness or pain thresholds will be defined by asking them to verbally rate their bothersomeness or pain using a 0–10 (0, no bothersomeness or pain; 10, intolerable bothersomeness or pain) numerical rating scale (NRS). The electrical current intensity (mA) of the electrical stimulator and the cuff pressure (mmHg) of the cuff inflator system will be set to target a bothersomeness or pain intensity of about 4 on the NRS
2. In the imaging session, fMRI and structural data will be acquired for the steady-state resting scan run (REST), event-related block design bothersomeness and emotion stimuli model scan run (TASK 1), structural image acquisition using three-dimensional T1-weighted turbo field echo (3D-T1W), event-related block design bothersomeness and emotion stimuli model scan run (TASK 2), diffusion tensor imaging (DTI), and the steady-state LBE pain-model scan run (TASK 3)
  Both TASK 1 and 2 will consist of a block design with 20 6-s bothersomeness and emotion stimulation blocks, which will be followed by 9-s inter-stimulus interval (ISI) blocks in order to prevent anticipation during the measurements. The 20 stimuli of bothersomeness and emotion will be comprised of five types of stimuli (bothersomeness only, emotion (neutral) only, emotion (negative) only, bothersomeness and emotion (neutral), bothersomeness and emotion (negative)) and arranged in pseudo-random order. Bothersome stimuli will be delivered with the Stimuplex® HNS 12 electrical stimulator onto the low back of the ipsilateral side of the sciatica using the StiMus® surface electrode (HUREV Co., Ltd., Gangwon-do, Korea). Emotions will be elicited by utilizing stimuli from the standardized International Affective Picture System (IAPS) [39, 40], which has been frequently used in neuroimaging studies. IAPS pictures with human content will be used in the present study. Table 1 presents the reported valence and arousal of the 16 negative and 16 neutral IAPS pictures used. We selected pictures that have low differences in valence and arousal between sexes when there is often quite a difference according to gender in the IAPS pictures. The images will be projected through a goggle onto a screen in the MRI scanner. During the ISI and bothersome stimuli alone, a black screen will be displayed. The IAPS pictures will be shown for 6 s and in the combination of picture and bothersome stimuli, the bothersome stimulus will be delivered 3 s after the onset of the picture, based on studies on startle responses and physiological response times for emotional stimuli [41, 42].
  In TASK 3, the LBE model of endogenous LBP will be applied for the steady-state pain scan run. By inflating a cuff bladder made of rubber under the most painful area of the lower back, the extension angle of the lower back increases to induce pain. The cuff inflator (located in the control room) will be connected to the cuff bladder through a rubber tube so that the applied air pressure to the cuff bladder can be controlled according to patient response by manual operation outside the scanner.

**Table 1:** The means and standard deviations (SDs) for valence and arousal of the groups to the International Affective Picture System (IAPS) items used in the study

	Negative emotion		Neutral emotion	
	Male	Female	Male	Female
Valence, mean (SD)	2.66 (0.40)	2.28 (0.54)	5.11 (0.27)	5.08 (0.34)
Arousal, mean (SD)	4.65 (0.60)	5.12 (0.71)	3.43 (0.56)	3.47 (0.61)
IAPS sequence number	2141, 2205, 2455, 2590, 2700, 2750, 2799, 2800, 3220, 3230, 3350, 6311, 6570, 9220, 9265, 9415		2038, 2102, 2191, 2215, 2220, 2235, 2357, 2383, 2393, 2396, 2397, 2410, 2445, 2487, 2495, 2499	

### fMRI and DTI data processing

The acquired fMRI and structural data will be processed with conventional analysis packages including FMRIB’s Software Library (FSL; http://fsl.fmrib.ox.ac.uk/fsl) AFNI (Analysis of Functional NeuroImages; https://afni.nimh.nih.gov/afni), and FreeSurfer (https://surfer.nmr.mgh.harvard.edu/) in data processing steps including physiological noise correction (3dretroicor, AFNI), head motion correction (mcflirt, FSL), skull stripping for fMRI data (BET, FSL) and structural data (mri_watershed, FreeSurfer), brain to brain registration (FLIRT and FNIRT, FSL) and statistical analysis.

### Outcome

#### Primary outcome measurement

The primary outcome measure is the bothersomeness of chronic sciatica as assessed by VAS [43]. To understand the impact of chronic sciatica on the patients, we decided to use the VAS for bothersomeness as the primary outcome measurement other than the VAS for pain intensity. Bothersomeness will be quantified using the VAS score as determined on a 100-mm horizontal line (0, no bothersomeness; 100, intolerable bothersomeness). Participants will be asked to mark the degree of bothersomeness due to sciatica experienced during the previous week at baseline (visit 2), before each treatment session (visits 2–9), and at each post-treatment follow-up visit (visits 10–11); the primary endpoint will be VAS for bothersomeness score at visit 10, i.e., 1 week after completion of the eight sessions of acupuncture treatment. The outcome measures will be assessed before treatment at each visit.

#### Secondary outcome measurements

VAS for pain intensity of chronic sciatica will be measured in the same fashion as VAS for bothersomeness (0, no pain; 100, intolerable pain) at baseline (visit 2), before each treatment session (visits 2–9), and at each post-treatment follow-up visit (visits 10–11). Since one of the goals of this study is to demonstrate the mechanism of acupuncture on chronic pain, we decided to use the VAS for pain intensity, for it is a fast and straightforward measure for assessing subjective intensity of pain [44].

The Oswestry Disability Index (ODI) [45] will be used to assess disability or dysfunction related to sciatica. The ODI consists of 10 questions on daily activities including: (1) experiencing general pain, (2) practicing self-care (e.g., washing, dressing), (3) lifting objects, (4) sitting, (5) standing, (6) walking, (7) sleeping, (8) travelling, (9) engaging in sexual activity if applicable, and (10) participating in social activities. Each item is rated on a 6-point scale (0–5), with lower scores indicating less pain-related disability. Participants will be asked to complete the validated Korean version of the ODI at baseline (visit 2) and at each post-treatment follow-up (visits 10–11).

The EuroQol 5-dimension (EQ-5D) [46] scale will be employed for health-related quality of life in patients with chronic sciatica. The EQ-5D consists of five dimensions including: (1) usual activities, (2) mobility, (3) pain/discomfort, (4) anxiety/depression, and (5) self-care. Each dimension is rated on a 1- to 3-point scale, with lower scores representing better health status. Participants will be asked to complete the validated Korean version of the EQ-5D at baseline (visit 2) and at each post-treatment follow-up (visits 10–11).

The Coping Strategies Questionnaire (CSQ) [47] will be used to evaluate the coping strategy for pain or acupuncture treatment. The CSQ is made up of 48 items across eight areas classified as either adaptive coping or maladaptive coping: (1) catastrophizing, (2) distractor behaviors, (3) self-instructions, (4) ignoring the pain, (5) reinterpreting the pain, (6) hoping, (7) faith and praying, and (8) cognitive distraction. Each item is rated on a 7-point Likert scale. The CSQ will be administered at baseline (visit 2) and at each post-treatment follow-up (visits 10–11).

The Korean version of Beck’s Depression Inventory (BDI) [48] will be used to measure depressive symptoms at baseline (visit 2) and at each post-treatment follow-up (visits 10–11). The BDI is a 21-item self-administered questionnaire with each item having a 0–3 point response format and a maximum theoretical score of 63. The psychometric quality of this test is very good [49].

The State-Trait Anxiety Inventory (STAI) [50] will be used to evaluate anxiety as a psychological factor at baseline (visit 2) and at each post-treatment follow-up (visits 10–11). The STAI consists of 40 items including: 20 items on the state of anxiety, which is triggered by a specific event and 20 items on the trait of anxiety, which is derived from personal characteristics. Each item is rated on a 4-point Likert scale, with lower scores indicating a lower degree of anxiety.

### Adverse events

Any expected or unexpected adverse events (AEs) will be recorded and described as frequency and percentage by the participants and practitioners at every visit until completion. Participants will be given notice of any cautions and possible AEs before the fMRI scanning and the acupuncture treatment. The AEs known as being related to acupuncture treatment include local bleeding or pain at the acupuncture points, local redness or bruising, and itching and dizziness during treatment [51]. If any serious AEs occur, detailed events including the date of occurrence, measures taken related to the treatment, causal relationship with the treatment, and treatment of the AEs will be announced immediately to the principal investigator and the Institutional Review Board (IRB) and direct actions will be supplied to those involved.

### Sample size

Referring to a previous study [52], an appropriately powered full-scale sample size was estimated using the mean difference of VAS for bothersomeness due to sciatica between groups in the primary endpoint. The researchers expect the mean difference will be 12.4 mm with a standard deviation (SD) of 16.2 mm between the two groups, which is not moderately clinically meaningful but represents a minimally important difference [53].

Considering the mean comparison method of the two-sample *t* test model with a two-tailed test, 80% test power, and a 5% significance level, the total number of individuals required per group is 27. For equal allocation of the two groups, the total sample size required when considering a dropout rate of 20% is 68 subjects with 34 subjects in each group.

Considering the nature of fMRI studies that use approximately 90,000 voxels to estimate the BOLD signal indirectly and for which conventional power calculations are meaningless, we determined 20 subjects in each group for fMRI scanning according to the trend of related neuroimaging studies [54–58]. Most studies decided that 20 participants is an adequate sample size regarding the high cost of fMRI examinations.

### Statistical analysis

All analyses will be performed using Statistical Package for Social Science (SPSS) for Windows (version 18.0; IBM Corp., Armonk, NY, USA) by a statistician blinded to the allocation of groups and the significance level will be set at 0.05. Statistical analyses will be conducted using the principle of the intention-to-treat (ITT) and the per-protocol (PP) analysis. We will apply the multiple imputation analysis for ITT analysis.

A paired *t* test (parametric) or Wilcoxon signed-rank test (non-parametric) will be used to compare continuous variables within groups. The two-sample *t* test (parametric) or the Mann-Whitney *U* test (non-parametric) for quantitative data and chi-square test or Fisher’s exact test for qualitative data will be performed to test differences between groups. Analysis of covariance (ANCOVA) will be used for adjustment of baseline characteristics if there is possibility of covariance. Repeated measure analysis of variance (ANOVA) will be performed for the different time point assessments between groups and interaction between groups and observed time. A mixed-model approach will also be used if necessary.

Statistical analysis for fMRI data will be completed via a general linear model (GLM) analysis (FEAT, FSL) and probabilistic independent component analysis (pICA using MELODIC, FSL). In performing GLM analysis, estimated responses from estimating regressors including stimulus function (block design) and nuisance regressors will be compared using a *t* test with the time series data in each brain voxel so that we can identify the specific brain responses to the visual and bothersome stimulus in TASK 1 and 2. pICA will be applied for fMRI data from REST and TASK 3 runs for brain functional connectivity analysis. In pICA processing, it assumes that the intrinsic sources in the data are non-Gaussian and the data can be inferred by Bayesian estimation of Gaussian noise. Then, the data can be projected in lower dimensional subspace and we can estimate the independent intrinsic component maps.

### Data collection, management, and monitoring

The data will be collected through paper-based documents written by the clinical research coordinator and outcome assessors. All original data sources will be stored in limited-access areas for 3 years. A clinical research associate independent from the funder and competing interests will monitor the clinical trial during the study period. The clinical research associate will monitor the written informed consent documents, recruitment status, protocol compliance, overall trial progress, data quality, timelines of data collection, treatment administration, and other relevant trial aspects and processes. The Ministry of Food and Drug Safety (MFDS) in Korea will carry out audits at regular intervals.

## Discussion

A systematic review of acupuncture for treating sciatica found that the use of acupuncture may be more effective than drugs and may enhance the effect of drugs for patients with sciatica, but because of the insufficient number of relevant and rigorous studies, the evidence is limited [20]. Among the included 11 studies, six used EA, two used manual acupuncture, and three used warming acupuncture as intervention. We adopted a complex intervention of manual acupuncture plus EA treatment because it is the most common approach used by DKMs in South Korea today.

Recently, neurophysiological studies for chronic pain are increasing [15–19] because emotional state, perception, modulation, and chronification all play an extremely important role in chronic pain [28, 29]. Kaneko et al. [59] demonstrated that the presence of psychiatric problems was associated with attenuated activity of the nucleus accumbens (NAc) in chronic LBP and dysfunction of the NAc might potentially be involved in the affective/motivational factors in the chronification of LBP. Thus, we designed a study protocol to examine the effect of acupuncture on parameters of emotion and coping strategy with neuroimaging as well as bothersomeness and pain intensity in patients with chronic sciatica.

In the fMRI scanning session, both TASK 1 and 2 were designed to investigate the interaction between negative and neutral emotional states and experimental bothersomeness in the essential pain-processing network [60]. As we decided to use the VAS for bothersomeness as the primary outcome measurement other than the VAS for pain intensity to understand the impact of chronic pain on patients in this study, we adopted this bothersomeness and emotion stimuli model scan run using the StiMus® MRI-compatible surface electrode to induce experimental bothersome stimuli other than pain stimuli. In TASK 3, we selected the LBE pain model because it is intuitive and has a simple methodology, induces a similar pain sensation to clinical pain in patients, and is a MRI-compatible pain model for neuroimaging study [61, 62]. The fMRI data analysis will be conducted to compare not only the acupuncture group with sham control group but will also compare responders with non-responders.

We chose non-penetrating sham acupuncture plus placebo acupuncture without electrical stimulation at non-traditional acupuncture points for the control group. Park et al. validated the complete subject blinding of sham acupuncture as the control group [38], and we will evaluate the blinding index [33] of the acupuncture and sham acupuncture treatments after the first acupuncture treatment and at endpoint. For valid blinding of placebo EA, the ES-160 dummy device was designed to have no electrical stimulation by insulating electrodes, similar to a previous study [63]. The intensity of electrical stimulation from both real and sham devices will be maintained under a certain level so that subjects in either group cannot perceive the electric current. Patients will be informed about the two types of acupuncture in the study as follows: “In this study, different types of acupuncture will be compared. The electric current will flow but with intensity under a fixed level which you cannot detect.”

This randomized, patient-assessor blind, sham-controlled clinical trial should provide important evidence for the efficacy of acupuncture for chronic sciatica and will help improve our understanding of the neurophysiological mechanism of acupuncture on the interaction between emotional state and chronic pain. The Standard Protocol Items: Recommendations for Interventional Trials (SPIRIT) Checklist for this protocol is attached in Additional file 2.

## Study limitations

Due to the nature of clinical research on acupuncture, practitioner blinding is impossible. Instead, we adopted the design of patient-assessor blinding, the best way of masking in acupuncture studies. Also, there is no limit on gender for enrollment, though the IAPS pictures used in TASK 1 and 2 of fMRI scanning session often have quite a different valence and arousal according to the gender [39, 40]. Thus, we selected pictures that have low differences in valence and arousal between sexes and subgroup analysis will be conducted.

## Trial status

Recruitment began in March 2018.

## Additional files

Additional file 1: Standards for Reporting Interventions in Clinical Trials of Acupuncture (STRICTA). (DOC 55 kb)

Additional file 2: Standard Protocol Items: Recommendations for Interventional Trials (SPIRIT) Checklist. (DOC 138 kb)

## Abbreviations

3D-T1W: Three-dimensional T1-weighted
ACC: Anterior cingulate cortex
AE: Adverse events
AFNI: Analysis of Functional NeuroImages
ANCOVA: Analysis of covariance
ANOVA: Analysis of variance
BDI: Beck’s Depression Inventory
BOLD: Blood-oxygenation-level-dependent
CSQ: Coping Strategy Questionnaire
DKM: Doctor of Korean Medicine
DLPFC: Dorsolateral prefrontal cortex
DMN: Default mode network
DTI: Diffusion tensor imaging
EA: Electroacupuncture
EQ-5D: EuroQol five-dimensional questionnaire
fMRI: Functional magnetic resonance imaging
FOV: Field of view
FSL: FMRIB’s Software Library
GLM: General linear model
HIVD: Herniated intervertebral disk
IAPS: International Affective Picture System
IRB: Institutional Review Board
ISI: Inter-stimulus interval
ITT: Intention-to-treat
LBE: Low back extension
LBP: Low back pain
NAc: Nucleus accumbens
NRS: Numerical rating scale
ODI: Oswestry Disability Index
PP: Per-protocol
SD: Standard deviation
SOP: Standard operating procedure
SPIRIT: Standard Protocol Items: Recommendations for Interventional Trials
SPSS: Statistical Package for Social Science
STAI: State-Trait Anxiety Inventory
STRICTA: Standards for Reporting Interventions in Clinical Trials of Acupuncture
TE: Time to echo
TR: Time to repetition
VAS: Visual analog scale
