# Supplementary material for: Effect and neurophysiological mechanism of acupuncture in patients with chronic sciatica: protocol for a randomized, patient-assessor blind, sham-controlled clinical trial
Source: Trials. 2019 Jan 16;20:56. doi: 10.1186/s13063-018-3164-8 (PMC6335765; doi:10.1186/s13063-018-3164-8)
Supplement: Supplementary file 1 — Standards for Reporting Interventions in Clinical Trials of Acupuncture (STRICTA). (DOC 55 kb) [file 13063_2018_3164_MOESM1_ESM.doc]

**Appendix 1. Acupuncture treatment details as recommended by STRICTA**

| Item | Details |
| --- | --- |
| 1. Acupuncture rationale | 1a) Style of acupuncture  - Manual acupuncture and electroacupuncture based on traditional meridian theory |
|  | 1b) Reasoning for treatment provided, based on historical context, literature sources, and/or consensus methods, with references where appropriate  - on the basis of a literature review regarding acupuncture for low back pain and sciatica [1-4]  - by a process of consensus with participating 12 doctors of Korean Medicine who specialize in Rehabilitation Medicine of Korean Medicine at several offline meetings |
|  | 1c) Extent to which treatment was varied  - Partially individualised acupuncture treatment, i.e., fixed essential points plus additional individualized points chosen by the practitioners among the predefined group of optional points categorized according to the three types of meridian patterns (Gallbladder meridian pattern, Bladder meridian pattern, and Mixed pattern) |
| 2. Details of needling | 2a) Number of needle insertions per subject per session (mean and range where relevant)  - From 7 to 15 |
|  | 2b) names (or location if no standard name) of points used (uni/bilateral)  - Seven fixed essential points: GB30, Ex-B2 at L4 and L5 (0.5 cun lateral to the lower border of L4-L5 spinous process [5]), BL23, BL25, BL40, and GB34 (unilateral)  - Eight or less additional individualized points : according to the three types of meridian patterns   1. Gallbladder meridian pattern: GB12, GB26, GB41 2. Bladder meridian pattern: BL24, BL37 3. Mixed meridian pattern: ST4, ST36, SP13, SP14, GV3, GV4, GV5, GV24, GV26 |
|  | 2c) Depth of insertion, based on a specified unit of measurement or on a particular tissue level  - From 5 to 20 mm |
|  | 2d) Response sought (for example, de qi or muscle twitch response)  - De qi sensation |
|  | 2e) Needle stimulation (manual, electrical)  - Manual stimulation: needle rotation with thumb and index fingers at 3 Hz to one fixed essential points of GB30 and eight or less additional individualized points  - Electrical biphasic waveform current: biphasic waveform current, which utilizes alternating interrupted wave and a continuous wave, in triangular form, at a frequency of 50 Hz by electronic stimulator (ES-160, ITO Co. Ltd., Tokyo, Japan) to six fixed essential points (except GB30) of Ex-B2 at L4 and L5, BL23, BL25, BL40, and GB34 (unilateral) |
|  | 2f) Needle retention time  - 15 min |
|  | 2g) Needle type (diameter, length, and manufacturer or material)  - A sterilised stainless steel needle (0.25 mm diameter × 40 mm length, Dongbang Acupuncture Inc., Korea) |
| 3. Treatment regimen | 3a) Number of treatment sessions  - Eight |
|  | 3b) Frequency and duration of treatment sessions  - 2 sessions/week for four weeks |
| 4. Other components of treatment | 4a) Details of other interventions administered to the acupuncture group (moxibustion, cupping, herbs, exercises, lifestyle advice)  - At the time of the first acupuncture session, all participants (both acupuncture group and sham control group) will be given an “exercise manual for patients with sciatica”.  - No other interventions during the study period allowed. |
|  | 4b) Setting and context of treatment, including instructions to practitioners, and information and explanations to patients  - Participants will be informed that the acupuncture treatment is based on Traditional Korean Medicine. |
| 5. Practitioner background | 5) Description of participating acupuncturists (qualification or professional affiliation, years in acupuncture practice, other relevant experience)  - Doctors of Korean Medicine (DKMs) who have at least three years of clinical experience after being certified with DKM licensure by the Korean Ministry of Health and Welfare and who specialize in Rehabilitation Medicine of Korean Medicine  - They will be required to take the educational course to strictly adhere to the study protocol and be familiar with administering study treatments. The techniques for the entire treatment procedures will be standardized between practitioners. |
| 6. Control or comparator interventions | 6a) Rationale for the control or comparator in the context of the research question, with sources that justify this choice  - Non-penetrating sham acupuncture (Park sham acupuncture) as control for manual acupuncture  - Placebo acupuncture without electrical stimulation as control for electroacupuncture  - Rationale of above controls is described in discussion of the manuscript [6, 7]. |
|  | 6b) Precise description of the control or comparator. If sham acupuncture or any other type of acupuncture-like control is used, provide details as for items 1 through 3 above  - From 7 to 15 non-traditional acupuncture points: 2 cm lateral to the each acupuncture point of real acupuncture group  - Except of the usage of a semi-blunted needle, the technique, needle retention time, treatment period, and number of treatment sessions will be the same as performed in the real acupuncture group.  - Non-penetrating disposable sterile stainless steel sham needles (0.25 mm diameter × 40 mm length, AcuPrime Co., Ltd, Exeter, UK) as described by Park et al. [6] and the ES-160 dummy device which is designed to have no electrical stimulation through insulation of electrodes will be used. |

[1] Committee on Compilation of Textbook in Society for Acupuncture & Moxibustion. Acupuncture and Moxibustion Medicine. Paju: Jipmoondang Publishing Company; 2012.

[2] Heo J, Ahn S, Kim N, Jeong C and Cha W. Donguibogam. Seoul: Ministry of Health & Welfare; 2012.

[3] Qin Z, Liu X, Wu J, Zhai Y and Liu Z. Effectiveness of Acupuncture for Treating Sciatica: A Systematic Review and Meta-Analysis. Evid Based Complement Alternat Med. 2015;2015:425108; doi: 10.1155/2015/425108 [doi].

[4] Yuan J, Kerr D, Park J, Liu XH and McDonough S. Treatment regimens of acupuncture for low back pain--a systematic review. Complement Ther Med. 2008;16:295-304; doi: S0965-2299(08)00043-5 [pii].

[5] Cabioglu MT and Arslan G. Neurophysiologic basis of Back-Shu and Huatuo-Jiaji points. Am J Chin Med. 2008;36:473-9; doi: S0192415X08005916 [pii].

[6] Park J, White A, Stevinson C, Ernst E and James M. Validating a new non-penetrating sham acupuncture device: two randomised controlled trials. Acupunct Med. 2002;20:168-74.

[7] Chen ZX, Li Y, Zhang XG, Chen S, Yang WT, Zheng XW, et al. Sham Electroacupuncture Methods in Randomized Controlled Trials. Sci Rep. 2017;7:40837; doi: 10.1038/srep40837 [doi].
